# Nurses’ intention to leave their job and associated factors in Bahir Dar, Amhara Region, Ethiopia, 2017

**DOI:** 10.1186/s12912-020-00439-5

**Published:** 2020-06-08

**Authors:** Emiru Ayalew, Yinager Workineh

**Affiliations:** 1 Department of Adult Health Nursing, College of Medicine and Health Science, Bahir Dar University, Bahir Dar, Ethiopia; 2 Department of Child Health Nursing, College of Medicine and Health Science, Bahir Dar University, Bahir Dar, Ethiopia

**Keywords:** Intention to leave, Nurse, Factors, Ethiopia

## Abstract

**Background:**

Intention to leave is an employee’s plan of tendency to leave the current working institute to find an alternative job in the near future. Even though nurses are the backbone of patient caring, there was no study done on intention to leave their job in North West Ethiopia. Therefore, the aim of this study was to assess nurses’ intention to leave their job and associated factors in Bahir Dar, North West Ethiopia, 2017.

**Methods:**

An institutional-based cross-sectional study was conducted from 1st March to 30th March 2017. After proportional sample size allocation, 210 participants were selected by simple random sampling method. Data were collected by using a self-administered structured questionnaire. Statistical Package for Social Science version 23.0 was used to enter, clean, code and analyze the collected data. The association between independent and dependent variables was assessed by using bivariable and multivariable logistic regression model. Factors that had statistically significant association with the dependent variable (*P* < 0.05) were identified as significant in the multivariable logistic regression analysis.

**Result:**

From a total of 210 nurses, 191 of them were participating in this study making a response rate of 90.95%. From all nurses, 64.4%of them were employed in the hospital. In this study, nurses’ overall intention to leave their job was 64.9% (95% CI: [57.6, 71.2]). Nurses’ intention to leave their job was determined by disagree in recognition (AOR = 4.83; 95%CI: [1.73, 13.50]), and work itself (AOR = 31.30; 95%CI: [7.16, 136.78]).

**Conclusion:**

Nurses’ intention to leave their job in the current study was high. The contributing factors for this problem were disagree in recognition at work and work itself. Hence, we recommended that hospital and health center managers should maintain recognition at work and work itself to retain nurses.

## Background

Intention is a mental process or activity that represent a binder to carrying out an action in the future [1]. Intention to leave is defined as one’s behavioral attitude to leave the institution [2]. It is also defined as an employee’s plan of tendency to leave the current working institute to find an alternative job in the near future [3–5]. Even if intention to leave does not essentially mean actual worker departure, it is a strong forecast of actual staff resigned or the level to that a member anticipates going the connection with the current community or employer [6–8].

Even though health care requires a more skilled work today as a result of advancement in medical technology for more sophisticated patient care [9], nurses’ intention to leave their profession is a major problem or drawback worldwide particularly in Africa [10–12]. This problem brings losing of competent and qualified nurses [10–12]. As a result of such events, the shortage of nurses have created a health care crisis in developing countries by adversely affecting the quality of nursing care [13].

Several studies had shown a variable level of nurses’ intention to leave their profession across the globe. A study conducted in the Midwestern region of the United States revealed that nurses had a high level of intention to leave their profession [14]. The similar studies carried out in Tanzania, Malawi and South Africa indicated that 18.8, 26.5 and 41.4% of health workers had the intention to leave their job to seek employment elsewhere respectively [15].

The main reason for nurses’ intention to leave their job is to change the organization or profession and look forward new direction [16]. The evidence in Northern Italy stated that low job satisfaction, age ≥ 40 years, and part-time schedule increased nurses’ intention to leave their job [17]. Similarly, the study conducted in European countries pointed out that newly qualified and nearly retirement age nurses were more likely to leave their job [18]. Another previous study also indicated that the level of salary, availability of transport service, organizational policy, job dissatisfaction, working environment, work pressure and demographic factors was directly associated with nurses’ intention to leave their profession [14, 19–21].

Nurses’ intention to leave their profession has a significant impact on the performance, stability and productivity of the health facility. Thus, this issue should be received considerable attention worldwide specifically in developing countries since the evidence is scarce in such areas.

Assessing nurses’ intention to leave their job is very necessary to plan nurse’s retention mechanisms in the Ethiopian context. This study is also very important to add evidence for policy planners and program managers to improve such problems. Moreover, there were little or no studies done before on the issue of nurses’ intention to leave their job in Ethiopia. Therefore, the purpose of this study was to assess the nurses’ intention to leave their job and associated factors in Bahir Dar city, Amhara region, Northwest Ethiopia, 2017.

## Methods

### Study design, setting and period

The study was conducted in Bahir Dar public health institutions by using institutional-based cross-sectional study from 1st March to 30th March 2017. Bahir Dar is the capital city of the Amhara National, Regional State. It is located 565 km away from Addis Ababa (the capital city of Ethiopia) in the Northwest part of Ethiopia. It is the most beautiful city in Ethiopia that was awarded the prestigious UNESCO Cities Prize [22].

The public health centers in the city were Bahir Dar, Han, Shimbit, Shum Abo, Abay Mado, Zegie, Zenzelma, Tsiss Abay, Ginbot- 20 and Meshenti. Felege Hiwot and Addis Alem hospitals were the only public hospitals in this area. In addition to public health facilities, there were private hospitals, clinics, pharmacies and drug stores. According to the report of 2017, 8 MSc, 270 BSc and 163 diploma nurses were employed in Bahir Dar public health facilities.

### Sample size determination and sampling procedure

The sample size was determined using a single population proportion formula. In this sample size calculation, 95% confidence intervals (CI) with Z α/2 value of 1.96, 60.9% proportion (p) [23], 5% margin of error (d) and 5% non-response rate were applied assumptions in this study.

$$ \mathrm{N}=\frac{{\left({\mathrm{z}}_{\upalpha /2}\right)}^2\mathrm{p}\left(1-\mathrm{p}\right)}{{\mathrm{d}}^2} $$

$$ \mathrm{N}=\frac{(1.96)^2\mathrm{0.0.609}\left(1-0.609\right)}{(0.05)^2}=365.9=366 $$

After the application of correction formula and 5% non-response rate on the above value (366), the final sample size was 210.

**Fig. 1:**
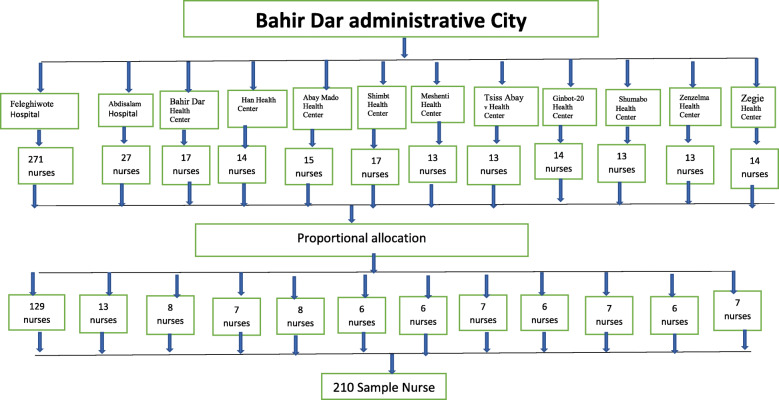
schematic presentation of sampling procedure for the study area, Bahir Dar, Ethiopia, 2017

All public hospitals and health centers in Bahir Dar were included in the study. From each selected public health institutes, the number of nurses were gotten from monthly payrolls. Then, based on the number of nurses in each facility, the sample size was proportionally allocated by using “n = (n/N) *ni” formula (Fig. 1).

Finally, simple random sampling method was carried out to select the study participants from all categories of nurses in each health institution. We excluded nurses in annual, study, and maternity leave from the study participants.

### Measurement tool

Data were collected by using pre-tested structured self-administered questionnaire. This questionnaire was developed from previous different researches. The consistency of the questionnaire was ensured by translated the English version into the Amharic version and then back to English with the same expertise. Correction and modification of the questionnaire was performed after a pre-test on 5% of the total sample size. Three BSc nurses and one experienced public health officer were selected as data collectors and supervisor respectively. Two days training was given to both data collectors and supervisor on the content of tools, and how to approach participants.

The socio-demographic characteristics and institutional factors were independent variables in the current study. The socio-demographic variables were age, work experience, level of education, and type of institution, whereas institutional factors were achievement, advancement, recognition at work, work itself, growth at work, organization policy, relationship with friends, relationship with supervisors, work security, payment and working conditions.

A 5-point Likert scale (strongly agree =5, agree =4, neither agree nor disagree =3, disagree =2, strongly disagree =1) was used to measure institutional factors [24, 25]. The response to these variables was categorized as agreed if the score > =mean, and disagree if the score is below the mean.

Nurses’ intention to leave their job, the dependent variable, was evaluated by Mark C Hand tool [26]. Seven items with a 5-point Likert were used in this instrument. The 5-point Likert scale ranged from 1 (strongly disagree) to 5 (strongly agree). Then the scores are categorized in low intent to leave (7 to 15), uncertain about intent to leave (16 to 25), and high intent to leave (26 to 35). Finally, the demarcation threshold formula: {(total highest score-total lowest score) / two} + total lowest score [27] was used to classify the outcome variables as intended to leave and unintended to leave.

### Data processing and analysis

Statistical Package for Social Science (SPSS) version 23.0 was used to enter, clean, code and analyze collected data. Frequency distribution was performed. The association between independent and dependent variables was assessed by using bivariable and multivariable entered logistic regression model. In this model, the odds ratio with a 95% confidence interval was used to determine the strength of the association between dependent and independent variables. Those variables, with *P* < 0.2 in the bivariable analysis model, were transferred to multivariable. Finally, factors that had statistically significant association with the dependent variable (*P* < 0.05) were identified as significant in the multivariable logistic regression analysis.

## Results

### Socio-demographic characteristics

One hundred ninety-one nurses participated in the study, making a response rate of 90.95%. Among all nurses, 105 (55%) were males. The mean age of nurses was 29.92 (SD ± 6.182) years. Regarding to marital status, 110(57.6%) of them were married. From all category of nurses, 100 (55.7%) were BSc nurses. From all participants, 62(34.3%) of them had 2–5 year of work experience. In the current study, the majority (64.4%) of nurses were employed in the hospital (Table 1).

**Table 1:** socio-demographic characteristics of study participant at Bahir Dar, Amhara Region, Ethiopia, 2017(*N* = 191)

Variable	Category	Frequency	Percent
Sex	Male	105	55
	Female	84	45
Current age in year	20–30	126	66.3
	31–40	53	27.1
	41–50	11	6.1
	> 51	1	0.6
Marital status	single	54	28.3
	Married	110	57.6
	Divorce	14	7.3
	Widowed	13	6.8
Religion	Orthodox Tewahedo	113	59.2
	Protestant	31	16.2
	Muslim	24	12.6
	Other	23	12.0
Ethnicity	Amhara	166	86.9
	Oromo	11	5.8
	^a^Other	14	7.3
Educational status	Diploma nurse	79	41.4
	BSc nurse	108	56.5
	MSc	4	2.1
Work experience	< 2 year	73	38.2
	2-5 year	34	17.8
	5–10 year	38	19.9
	> 10 year	46	24.1
Institution	Hospital	123	64.4
	Health center	68	35.6

^a^ Tigrie, GUmuze, Kembata, Sidama

### Level of nurses’ intention to leave their job

From all participants, 64.9% (95% CI: [57.6, 71.2]) of them had intention to leave their job. Among the study participants who had the intention to leave, 53.4% of them had a high level of intention to leave their job (Table 2).

**Table 2:** Nurses intention to leave their job in Bahir Dar, Amhara Region, Ethiopia, 2017(*N* = 191)

Variables	Responses	Frequency	Percent
Nurse intention to their job	Intention to leave	123	64.6
	Not intentional to leave	68	35.4
Levels of intention to leave	Highly intended	66	53.4
	Uncertain	47	38.7
	Low intended	10	7.9

### Factors associated with nurses’ intention to leave their job

After application of multivariable logistic regression model, only work itself and recognition at work was the main determinants of nurses’ intention to leave their job. For instance, those study participants who disagreed with work itself in their organization were 31.30 times (AOR: 31.30; 95%CI: [7.16, 136.78]) more likely to leave their job as compared their counterparts. Similarly, those study participants who disagreed in the recognition given to their work had 4.83 odds (AOR: 4.83; 95%CI: [1.73, 13.50]) of intention to leave their job than their counterparts (Table 3).

**Table 3:** Factors associated with intention to leave their job in Bahir Dar, Amhara Region, Ethiopia, 2017(*N* = 191)

Variables	Categories	Level of Intention		OR (95% CI)	
		Intended to leave N (%)	Not Intended to leave N (%)	COR (95% CI)	AOR (95% CI)
Sex	Male	71(66.7)	34(32.4)	1.00	
	Female	53(61,6)	33(38.4)	1.3(0.72,2.36)	0.99(0.43,2.29)
Age	20–30	45(35.7)	81(64.3)		
	31–40	18(34)	35(66)		
	41–50	7(63.6)	4(36.4)		
	> = 51	0(00)	1(100)	1.00	
Work experience	< 2 year	52(71.2)	21(28.8)	1.45(0.66,3.18)	
	2-5 year	19(55.9)	15(44.1)	0.74(0.30,1.83)	
	5–10 year	24(63.2)	14(36.8)	1.10(0.41,2.45)	
	> 10 year	29(63)	17(37)	1.00	
Level of education	Diploma	49 (62)	30 (38)	0.54(0.05,5.48)	
	BSc	72(66.7)	36 (33.3)	0.67(0.07,6.64)	
	MSc	3(75)	1(25)	1.00	
Institution	Hospital	76(61.8)	47(38.2)	1.00	
	Health center	48(70.6)	20(29.4)	1.48(0.79,2.80)	1.28(0.48,3.40)
Achievement	Agree	70(56)	55(44)	1.00	
	Disagree	54(81.8)	12(18.2)	3.54(1.72,7.25) **	1.40(0.51,3.86)
Advancement	Agree	41(52.6)	37(47.4)	1.00	
	Disagree	83(73.5)	30(26.5)	2.59(1.36,4.59) **	1.52(0.68,3.41)
**Recognition at work**	**Agree**	**60(52.6)**	**54(47.4)**	**1.00**	
	**Disagree**	**64(83.1)**	**13(16.9)**	4.43(2.2,8.93) **	4.83(1.73,13.50) **
**Work it self**	**Agree**	**38(42.2)**	**52(57.8)**	**1.00**	
	**Disagree**	**86(85.1)**	**15(14.9)**	7.85(3.94,15.64) **	31.30(7.16,136.78) *
Growth at work	Agree	74(56.1)	58(43.9)	1.00	
	Disagree	50(84.7)	9(15.3)	4.35(2.20,8.93) **	2.49(0.88,7.00)
Organization policy	Good	34(48.6)	36(51.4)	1.00	
	Poor	90(74.4)	31(25.6)	3.07(1.65,5.72) **	0.21(0.04,1.11)
Relationship with friends	Agree	56(56)	44(44)	1.00	
	Disagree	68(74.7)	23(25.3)	2.32(1.26,4.30) **	2.69(.087,8.39)
Relationship with supervisors	Agree	42(55.3)	34(44.7)	1.00	
	Disagree	82(71.3)	33(28.7)	2.01(1.10,3.69) **	0.64(0.25,1.68)
Work security	Agree	21(41.2)	30(58.8)	1.00	
	Disagree	103(73.6)	37(26.4)	3.98(2.03,7.79) **	2.70(0.87,8.38)
Payment	Agree	53(64.6)	29(35.4)	1.00	
	Disagree	71(65.1)	38(34.9)	1.02(0.56,1.86)	0.07(0.01,1.70)
Working condition	Agree	43(60.6)	28(39.4)	1.00	
	Disagree	81(67.5)	39(32.5)	1.35(0.74,2.50)	7.61(0.72,80.82)

* P<0.05, **P<0.005

## Discussion

The current study was aimed to assess nurses’ intention to leave their job and associated factors by using a cross-sectional study in Bahir Dar North West Ethiopia. Focused on such objective, 64.9% of nurses hand the intention to leave their job in the current area. This finding is consistent to the studies conducted in Jeddah City (61.5%) [28], Jordan (60.9%) [12], Jimma zone (63.7%) [29] and East Gojjam zone (59.4%) [23].

On the other hand, the present finding is higher than studies done in Gondar (52.5%) [30] and Sidama (50%) [10]. The reason for such difference might be variation in health facility and categories of health professions in the current and previous study sites. Our study participants are nurses from both hospitals and health centers, whereas the previous study participants were all health workers from hospitals. Similarly, the studies conducted in Tanzania (18.8%), Malawi (26.5), South Africa (41.4%), Saudi Arabia (40%), Macao (39%) and Iran (21%) [15, 17, 31–33] revealed that the proportion of nurses’ intention to leave their job was lower than the current study. The presence of ongoing interventions, attractive salary (incentives), and better infrastructures in the above countries [34–37] brings lower level of nurses’ intention to leave their job than in the present site.

This finding indicated that the demographic characterstics such as sex and mean age of the participants were different from the studies carried out in the other corner of the globe. The mean age of the participants this study was 29.92 years that is younger than studies done in Rwanda, Turkey, and Iran [38–40].

In the current study, 53.6% of nurses were male which are similar to the study done in Wollega Ethiopia [41]. On the other hand, the current finding is higher than the study conducted in the United States (7%) [42] and Iran 12.7% [39]. The main reason for Ethiopia male to join the nursing profession is to easily fix their job as compared with other professions.

In the current study, the main determinants of nurses’ intention to leave their job was recognition at work and work itself. Nurses who disagreed on the recognition given in their workplace were more likely to leave their job than their counterparts. This finding is consistent with the studies done in India, Malaysia, China, Saudi Arabia, Sweden, Tigray, Bangladesh, and Pakistan [31, 43–48]. The reason for a high level of nurses’ intention to leave their job in this regard was due to lack of acknowledging the employees’ efforts and their accomplishments through praise, respect, and thanks [49–54]. The other evidence also pointed out that nurses intended to leave their job unless they received support from their managers [55]. Hence, constructive recognition should be given to promote nurse’s retention and provide better care to patients.

Similarly, the current finding stated that participants who disagreed on the work itself in their organization had more fold of intention to leave their job as compared with their counter parts. This finding was in line with studies conducted in New Delhi (India), Malaysia, and China [43, 56, 57]. The main reason for a high level of intention to leave among participants who disagreed on the work itself in their institution is as a result of the negative impact of work satisfaction on nurses’ motivation and organizational profitability [58]. The other evidence also stated that the work itself harms worker retention [31, 59].

## Conclusion

Nurses’ intention to leave their job in the current study was high. The contributing factors for this problem were disagree in recognition at work and work itself. Hence, we recommended that hospital and health center managers should maintain recognition at work and work itself to retain nurses.

## Supplementary information

**Additional file 1.** Questionnaire.

## Data Availability

Data of this study will be obtained by contacting the corresponding author via this email: emiruayaew2010@gmail.com or Tel no. + 251912121688.

## Abbreviations

AOR: Adjusted odds ratio
CI: Confidence interval
COR: Crowd Odd Ratio
FHRH: Felege Hiwot referral hospital
HC: Health Center
OR: Odds ratio
P: Prevalence
SPSS: Statistical Package for Social Science
