# Supplementary material for: Nurses’ intention to leave their job and associated factors in Bahir Dar, Amhara Region, Ethiopia, 2017
Source: BMC Nurs. 2020 Jun 8;19:46. doi: 10.1186/s12912-020-00439-5 (PMC7278137; doi:10.1186/s12912-020-00439-5)
Supplement: Supplementary file 1 — Additional file 1. Questionnaire. [file 12912_2020_439_MOESM1_ESM.docx]

**Additional file 1: Questionnaire**

PART I : **Socio Demograhic Characterstics**

Instructions: if you are voluntary to participate in the study please answer the following questions about yourself.

1. Age _______
2. sex: female male
3. ethnicity: Amhara Oromo Tigray if others specify_______
4. Level of education in nursing: diploma bachelor degree MSc
5. for how many years have you been working with this health facility? --------------
6. marital status: Single Married Divorce Widow
7. Which health institution could you work? Hospital Health center
8. Religion Orthodox Tewahido Muslim Protestant Catholic If others specify—

**PART II: measure of hygiene and motivational factors.**

This section of the questionnaire asks for your opinion about whether you agreed or not at this facility. Please circle the one number for each question that comes closest to reflecting your opinion about it. (strongly agree (SA) =5, agree (A) =4, neither agree nor disagree (N) =3, disagree (D) =2, strongly disagree (SD) =1)

| Motivation  Factors | S.n | Statement | Possible answer | | | | |
| --- | --- | --- | --- | --- | --- | --- | --- |
|  |  |  | **SD** | **D** | **N** | **A** | **SA** |
| Achievement | 109 | I am proud to work in this health institution because it recognizes my achievements. | 1 | 2 | 3 | 4 | 5 |
|  | 110 | I feel satisfied with my job because it gives me feeling of accomplishment. | 1 | 2 | 3 | 4 | 5 |
|  | 111 | I feel I have contributed towards my health institution in a positive manner. | 1 | 2 | 3 | 4 | 5 |
|  | 112 | I will choose career advancement rather than monetary incentives. | 1 | 2 | 3 | 4 | 5 |
| Advancement | 113 | My job allows me to learn new skills for career advancement. | 1 | 2 | 3 | 4 | 5 |
| Work itself | 114 | My work is thrilling and I have a lot of variety in tasks that I do. | 1 | 2 | 3 | 4 | 5 |
|  | 115 | I am empowered enough to do my job. | 1 | 2 | 3 | 4 | 5 |
|  | 116 | My job is challenging and exciting. | 1 | 2 | 3 | 4 | 5 |
| Recognition | 117 | I feel appreciated when I achieve or complete a task. | 1 | 2 | 3 |  |  |
|  | 118 | My manager always thanks me for a job well done. | 1 | 2 | 3 | 4 | 5 |
|  | 119 | I receive adequate recognition for doing my job well. | 1 | 2 | 3 | 4 | 5 |
|  | 120 | I am proud to work in my health institution because I  feel I have grown as a person | 1 | 2 | 3 | 4 | 5 |
| Growth | 121 | my job allows me to grow and develop as a person. | 1 | 2 | 3 | 4 | 5 |
|  | 122 | My job allows me to improve my experience, skills and performance. | 1 | 2 | 3 | 4 | 5 |
| Hygiene factors | | Statement | **SD** | **D** | **N** | **A** | **SA** |
| Organization  Policy | 123 | The attitude of the administration is very accommodative in my health institution. | 1 | 2 | 3 | 4 | 5 |
|  | 124 | I am proud to work for this health institution because the health institution policy is favorable for its workers. | 1 | 2 | 3 | 4 | 5 |
|  | 125 | I completely understand the mission of my health institution. | 1 | 2 | 3 | 4 | 5 |
| Relationship  With colleague | 126 | It is easy to get along with my colleagues. | 1 | 2 | 3 | 4 | 5 |
|  | 127 | My colleagues are helpful and friendly. | 1 | 2 | 3 | 4 | 5 |
|  | 128 | Colleagues are important to me. | 1 | 2 | 3 | 4 | 5 |
| Work security | 129 | I believe safe working at my workplace. | 1 | 2 | 3 | 4 | 5 |
|  | 130 | I believe my job is secure. | 1 | 2 | 3 | 4 | 5 |
|  | 131 | My workplace is located in an area where I feel comfortable. | 1 | 2 | 3 | 4 | 5 |
| Relationship With supervisor | 132 | I feel my performance has improved because of the support from my supervisor. | 1 | 2 | 3 | 4 | 5 |
|  | 133 | I feel satisfied at work because of my relationship with my supervisor. | 1 | 2 | 3 | 4 | 5 |
|  | 134 | My supervisors are strong and trustworthy leaders | 1 | 2 | 3 | 4 | 5 |
| Payment | 135 | I am encouraged to work harder because of my Salary. | 1 | 2 | 3 | 4 | 5 |
|  | 136 | I believe my salary is fair. | 1 | 2 | 3 | 4 | 5 |
| Working  Conditions | 137 | I feel satisfied because of the comfort I am provided at work. | 1 | 2 | 3 | 4 | 5 |
|  | 138 | I am proud to work for my health institution because of the pleasant working conditions. | 1 | 2 | 3 | 4 | 5 |

**PART III. Intention To Leave Questionnaire**

This section of the questionnaire asks for your opinion about whether you intend to stay at this institution or not. Please circle the one number for each question that comes closest to reflecting your opinion about it.

| S. No | Statement | Rating | | | | |
| --- | --- | --- | --- | --- | --- | --- |
|  |  | **SD** | **D** | **N** | **A** | **SA** |
| 139 | All things considered; I would like to find a comparable job in a different organization | 1 | 2 | 3 | 4 | 5 |
| 140 | It is likely that I will actively look for a different organization to work for in the next year | 1 | 2 | 3 | 4 | 5 |
| 141 | At the present time I am actively planning to return to school | 1 | 2 | 3 | 4 | 5 |
| 142 | The results of my search for a new job is encouraging | 1 | 2 | 3 | 4 | 5 |
| 143 | I will probably look for a new job in the near future | 1 | 2 | 3 | 4 | 5 |
| 144 | At the present time, I am actively searching for a job in another organization | 1 | 2 | 3 | 4 | 5 |
| 145 | I intend to quit | 1 | 2 | 3 | 4 | 5 |
